# Supplementary material for: Using stochastic cellular automata to model and define sufficient conditions for the survival of Enterococcus faecalis biofilms with the pCF10 plasmid under erythromycin treatment
Source: PLoS Comput Biol. 2025 Sep 2;21(9):e1013425. doi: 10.1371/journal.pcbi.1013425 (PMC12445747; doi:10.1371/journal.pcbi.1013425)
Supplement: Attachment — Submitted filename: reviewPCOMPBIOL-D-24-02060v1.pdf [file pcbi.1013425.s037.pdf]

General comments:

To improve the reach of the paper I would highly recommend to re-structure the text. Overall, it was very difficult to read through and maintain interest, as the text appears to be out of order. Specifically, the paper would appeal to a wider audience, if it started by presenting the experimental basis for the model. It is stated multiple times that the goal of the paper is to model through a cellular automaton the restructuring of the biofilm after 1 hour. However, it is not until page 15 of the paper that any explanation of the actual biofilm restructuring is given, and even then, it is not clear to me, exactly what are the defining characteristics of the restructuring. Furthermore, figures are referenced out of order as a direct result of the current paper's structure.

From a readability point of view, the reader is required to remember countless mathematical terms, and these are not summarized in a table, which requires to scroll back and forth through the main text to remember the definitions. Furthermore, there is a lot of heavy mathematical language and definitions that might be difficult to understand for a broader audience, consider simplifying the notation and writing in plain English when possible.

Additionally, more justification is required about the assumptions in the model. As opposed to merely stating the rules of the simulation, it would be very helpful to explain why this are the appropriate assumptions. Similarly explain why cellular automata is the superior choice of simulation for this system.

In the supplement for figure 5 a graphPad project is provided, there is a lot of data and figures that are not explained anywhere. Please provide details, specifically data 6 looks very different from the data provided in the main text, please explain what this data correspond to. Overall, data provided for individual figures in supplements is lacking a read me or some explanatory text about what it is. Additionally, raw confocal images for the experimental data should be provided.

Specific comments:

1. Line 28: Consider re-writing sentence starting at Laboratory and ending in treatment. The intended meaning is not clear. Specifically, what do absorption buffer refer to?
2. Line 48: In contrast to what?
3. Line 64: What is the necessary and sufficient statement?
4. Line 75: Here would be a great place to explain exactly what the “demonstrated rapid one hour remodeling” is, providing a figure and accompanying diagram highlighting the specific characteristics that your model is looking to reproduce or explain.
5. Line 77: How does 2D capture the spatial heterogeneity of the biofilm? Can you please describe this heterogeneity? Please define which coordinates are being represented (xz), this information is provided later but it is confusing at this point. Could be part of the diagram suggested in comment 4.
6. Line 82: As I first read through, I was confused by the sentence “stressed cells require less room to divide”. I think this would be fixed by addressing comment 4, but overall please justify why “stressed cells require less room to divide”, and how “this difference in cellular division captures the biological process of aggregation”.
7. Line 86: Please address your choice of using cellular automata. Specifically, what are the advantages over a discrete agent-based model for example and why is this the correct model to represent the biofilm. Please provide possible limitations for your model.

8. Line 96: Please justify your choice for only 2 stress states. Additionally, in the diagram in fig 1. if a stressed cell divides shouldn't the new cell also be stressed, e.g. a member (1,1)? In a sense this goes back to my question about choosing a binary definition of stress.
9. Line 109: In definition 2, c is not defined anywhere in the text. Additionally when presenting definitions please wrtie the abbreviation next to the plain English. For example: the interaction neighborhood ($N_L$) of r for bacterial…
10. Line 121: delete recall that, it is likely not common knowledge for all readers.
11. Line 153-155: please define decay. Reference to fig 5 is out of order, will probably be solved by comment 4. Furthermore, how is your assumption that decay rate and death rate are equivalent. Can you measure decay rates from your experiments to verify this assumption?
12. Line 159: Would be good to move this to the description of your system in comment 4. It is a bit strange to specify micrometer units for L, without providing a typical numerical range.
13. Line 163: Please justify why 8 positions is sufficient and why diagonals or other possible configurations are not considered.
14. Line 177: Can you please explain why assuming a buoyant force makes sense in this system? One might think that capillary forces from the water and adhesion forces from the ECM would penalize out of plane growth and prefer side motion instead.
15. Fig 2. Caption "On the other hand, if $\vec{r}$ is to the left or right of $\vec{x}$ and if lattice nodes $\vec{y}_2$ or $\vec{y}_3$ are empty then $\vec{x}$ will not place the new cell in $\vec{r}$. $\vec{x}$ will instead place the cell in $\vec{y}_2$ or $\vec{y}_3$ due to the force of buoyancy." Are these labels correct? Should it be y1 or y2?
16. Line 183-192: Explain in plain English these probabilities please. Maybe consider simplifying the mathematical definitions to plain English and presenting the full mathematical framework in the SI.
17. Line 220: "It is known" please provide citation.
18. Line 221: First time that a rapid increase in bacterial population is mentioned, refer to comment 4.
19. Definition 7, same as before consider using plain English to explain the assumptions here. Please explain how $\psi$ and $\psi_2$ are determined.
20. Line 249, equation 10: Why is the probability of a cell being at stress 0 and remaining at stress 0 is equal to having a space remain empty?
21. Fig 3: Consider providing this diagram earlier, maybe as part of figure 1 explaining the experimental results and your proposed model.
22. Line 270: are cells non motile? Or can they move to other parts of the biofilm?
23. Line 282: provide citation for Todd-Hewitt broth.
24. Line 287: Define MIC
25. Table 1: Please provide variable names in plain English to refer to as reading the paper. Ta: how is this estimated?
26. Line 322: Provide definition of CFL acronym.
27. Line 373: How do you know that cells in protected regions are unstressed? I would suggest using a stress reporter on the experimental setup to verify this assumption if not done already.
28. Line 407-411 I don't understand this, can you please provide a diagram of the experimental setup to understand what is causing the described shift? Why is the system not allowed to equilibrate before imaging?

29. Line 421: In the current model only xz slices are considered, but does the region need to be protected in both x and y? I don't see islands of less-densely packed cells amongst dense structures in many of the images in Fig 5.
30. Lines 429- 438 do not belong in the results section.
31. Fig 5 caption: The number 72 doesn't add up. 12 x 3 replicates is 36?
32. Fig 5 and Line 449: before treatment there is a uniform lawn of cells, after treatment there is clear regions that no longer have cells, what happens to the cells in those regions? The increase in CFU might be related to relocation of the cells in those regions. Why are you confident that the treatment of Erythromicin does not affect the properties of the ECM such that the CFU counts are higher because it is easier to dissociate the structures, leading to more individual bacteria in the serial dilutions? Please provide a control image showing what a biofilm without pCF10 looks like before and after treatment, also please provide a control image showing what a treatment with only media looks like.
33. Line 445: "One can see that the amount of preformed .." As far as I can see, this is not shown, as preformed structures are not quantified anywhere.
34. Lines 451-453: please provide an image or diagram explaining this.
35. Line 464, make a reference to each individual panel at the different steps in the workflow please.
36. Fig 6.E, explain what the different colors in this image represent. Overall this image is missing labels and a clear description.
37. Fig 6.F Significance is provided with ** but caption doesn't specify what ** represents. Would be good to provide a panel with untreated and treated images in the supplement so the reader can visualize the difference in the protective volume.
38. Fig 6.G Please provide an example of how the protective region structure volume was calculated.
39. Fig 7, line 503-504. How do you know this is the eDNA cloud? Was the eDNA labelled separately? If not please justify or provide new labelled eDNA data.
40. Line 510: Overall, if parameters such as $\gamma_p$ are to be used far from their definition, please remind the reader what they stand for (or organize them all in an easy to reference table. See comment on table 1)
41. Line 521: Why does this boundary condition result in no flux through $\partial\Omega_1$?
42. Fig 8. Why is B not the same height (z) as A? It seems as if the biofilm is cropped?
43. Combine lines 534-536 and lines 543-544?
44. Please discuss if assumptions in your model, such as the buoyant force and limited motion of the cells (8 positions) could be responsible for the observed results in Fig 8.
45. Line 550- 552. This is not clear, please explain.
46. Can you estimate "CFUs" from your simulation results and match the doubling of cells in the experiment?
47. 577: Please explain why eDNA is not enough to protect WT E. faecalis. E.g what is the role of plasmid pCF10 in eDNA production. Without a control on WT, it's hard to interpret your results.
48. Line 591: Why is it not possible to use a constitutive reporter to label the cells, such that staining is not required? You state that the reporters affect the growth rate, but is this a big effect? Or is it a result of phototoxicity?
49. Please discuss other possible mechanisms other than eDNA that could lead to similar results
