# Supplementary material for: Using stochastic cellular automata to model and define sufficient conditions for the survival of Enterococcus faecalis biofilms with the pCF10 plasmid under erythromycin treatment
Source: PLoS Comput Biol. 2025 Sep 2;21(9):e1013425. doi: 10.1371/journal.pcbi.1013425 (PMC12445747; doi:10.1371/journal.pcbi.1013425)
Supplement: Attachment — Submitted filename: Response to reviewers.pdf [file pcbi.1013425.s038.pdf]

TEMPLE UNIVERSITY
College of Science and Technology

Department of Mathematics
Wachman Hall, Room 514
1805 North Broad Street
Philadelphia, PA 19122-6094

phone 215-204-2280
email queisser@temple.edu
web sites.temple.edu/queisser

May 6, 2025

Dr. Ricardo Martinez-Garcia
Academic Editor
PLOS Computational Biology

Revisions to Shoraka et al. (2025)

Dear Dr. Martinez-Garcia,

We would like to thank you and the reviewers for the constructive feedback to the manuscript “Using stochastic cellular automata to model and define sufficient conditions for the survival of Enterococcus faecalis biofilms with the pCF10 plasmid under erythromycin treatment” by Shoraka et al. We addressed all the comments that were raised by the reviewers and provide a point by point response attached to this letter and submitted a revised manuscript.

We look forward to hearing from you soon.

Best Regards,

Gillian Queisser

Professor
Department of Mathematics
Temple University
1805 N Broad Street
Philadelphia, PA 19122-6094, USA
https://sites.temple.edu/queisser/
phone: +1 (267) 294-1428

Reviewer #1

We thank the reviewer for their constructive feedback and have summarized our revision in a point by point way below.

* There is some (partially word-by-word) identity between abstract and introduction, would be great to have it not exactly word-by-word the same but adapted to the purpose, respectively.

We have rewritten the abstract to avoid any repetitions.

* As the model is in principle a hybrid setting (and not "only a CA"), one could mention that also in the title or at least in the abstract or so.

Thank you for the suggestion. A line has been added to the abstract reading: 'This model involves both stochastic cellular automata and deterministic partial differential equations.'

* Is it correct (following Fig. 1) - it is not possible in the setting to loose stress again?

Yes, that is correct. The induction of stress is, biologically, a cell's expression of aggregation substance. Aggregation substance is a sticky, large surface protein whose expression is an irreversible process. A more detailed explanation of this process is added between original lines 82-83 and new lines 293-298.

It reads: 'Biologically, the induction or switching from 0 to 1 of stress is the process of aggregation substance induction undertaken by the cell. Aggregation substance is a stable cell wall protein that binds lipoteichoic acid on adjacent cells and is an irreversible, discrete, and stable process [65]. A cell expressing aggregation substance is sticky and will not fully separate from any newly divided cell it produces thereby reducing the distance used for cellular division.'

* In the context of subsection 2.1.2: Have the antibiotics been represented on the same grid as the CA itself? Which means, the main difference between the antibiotics versus cells and stress level is that the first one can assume continuous values, whereas the second ones have the discrete states with 0 and 1? Has it been checked if that setting (including the time step size) would be ok concerning stability criteria for finite differences for the antibiotics?
(Maybe I have overlooked the concrete equations for the antibiotics, but if they are indeed not stated explicitly, this would be good to include, maybe in an appendix if it is too detailed for the main part of the paper)

Good question! Yes, they are on the same grid. In Section 3.4 the time step dt_a is computed to satisfy the stability requirements for the discretized antibiotic diffusion operator. This is the main reason for using a time splitting method when numerically simulating the equations since dt_a<<dt. Also, the PDE representing antibiotics has been added to the manuscript between lines 355 and 356 of the original submission.

* the within-figure-numbering in Fig. 2 is a bit complicated, with have two different subfigures A and B etc.

Good point! Figure 2 (now Figure 9, after having moved the results section prior to the Methods) has been edited to have clearer subfigure enumeration.

* in caption of Fig. 3, I think the "x-slice" should be typed with a small x, too, as directly above and around.

Yes, thank you. Corrected.

* at the beginning of the results part, section 3.1 (lines 429 ff), this is again doubling partially the

abstract. I think, would be good, which basics to introduce where, but once is enough (or at least not more or less identical)

Thank you for pointing this out! We have removed those pieces from Section 2.1.

Reviewer #2

Thank you for your detailed comments, we addressed each of them individually and have compiled our responses below:

General comments: To improve the reach of the paper I would highly recommend to re-structure the text. Overall, it was very difficult to read through and maintain interest, as the text appears to be out of order. Specifically, the paper would appeal to a wider audience, if it started by presenting the experimental basis for the model. It is stated multiple times that the goal of the paper is to model through a cellular automaton the restructuring of the biofilm after 1 hour. However, it is not until page 15 of the paper that any explanation of the actual biofilm restructuring is given, and even then, it is not clear to me, exactly what are the defining characteristics of the restructuring.

This is a good point! To make the paper more readable, we have moved the materials and models section to follow the results. Hopefully this helps with the flow of the paper!

Furthermore, figures are referenced out of order as a direct result of the current paper's structure. From a readability point of view, the reader is required to remember countless mathematical terms, and these are not summarized in a table, which requires to scroll back and forth through the main text to remember the definitions.

Great suggestion! We added a table listing all symbols we used in the mathematical formulation. The table appears on page 12. The figure referencing order has been corrected.

Furthermore, there is a lot of heavy mathematical language and definitions that might be difficult to understand for a broader audience, consider simplifying the notation and writing in plain English when possible.

This is a helpful suggestion! To make the paper flow better and appeal to a wider audience, we moved the results to follow the models and methods section. Additionally, plain English language was added to help readers understand the more technical terms the mathematics demand. Please see comments below on where these edits were made.

Additionally, more justification is required about the assumptions in the model. As opposed to merely stating the rules of the simulation, it would be very helpful to explain why this are the appropriate assumptions.

We thank the reviewer for pointing this out. In fact, the only assumption made that is not commonly held in biofilm modeling or standard chemical interaction modeling is that of growth anisotropy. The textbooks cited in Section 3.1 contain all background leading to the presented equations. The original statement on line 89 of the original submission (now line 312) has been modified to read: 'A detailed explanation of the framework on which this model is based can be found in [66-68].'

Please also note that the '8 positions' is not an assumption. It is a consequence of the mathematical discretization of the observed growth process and is compatible with the 'space' function of the model.

Throughout the paper, we have also added statements on the following lines of the original submission to justify certain observations and modelling choices more.

1. **82-83 (now lines 293-298)**
2. **107-108 (now line 306)**
3. **163 (now line 381)**
4. **Definition 7**
5. **Definition 2**

Similarly explain why cellular automata is the superior choice of simulation for this system.

Cellular automata and individual based models in general give the modeler unparalleled ability to capture fine-grain interactions between cells which is critical when exploring the cell-to-cell interactions that lead to a global response. Current work is being done to devise a continuum model, informed by the models developed in this paper, to run the simulations on larger, more complex domains. An explanation of why we use this model is included after line 86 of the original submission (now 306-312).

It reads: 'Previous attempts to directly construct a PDE model for bacterial growth with an associated stress response were futile as the cell-to-cell interactions were not well enough understood. Thus, we pivoted to using the framework of cellular automata which has given us unparalleled abilities to parse and validate cell-to-cell interactions that lead to the globally observed system behavior. The creation of this model is an important step towards constructing a continuum model with the intention of running simulations on larger, three-dimensional computational domains.'

An additional statement was added after line 614 of the original submission (now 719) stating the prohibitive time cost of running larger simulations (particularly those in 3D). We included the information that we are currently working on a continuum model which significantly reduces computational cost.

It reads: 'Mathematically, we are currently developing a continuum-level description of the cellular automata model to run simulations on larger, more complex computational domains. Cellular automata computationally scale poorly with increasing domain size, therefore a continuum model will be necessary.

In the supplement for figure 5 a graphPad project is provided, there is a lot of data and figures that are not explained anywhere. Please provide details, specifically data 6 looks very different from the data provided in the main text, please explain what this data correspond to.

Thanks for pointing this out! This has been fixed.

Overall, data provided for individual figures in supplements is lacking a read me or some explanatory text about what it is. Additionally, raw confocal images for the experimental data should be provided.

Raw confocal images were uploaded in the first submission (they are in the treated/untreated folders in the supplemental information). A readme file was also added to the supplemental information, hopefully this makes the code clearer!

Specific comments:

1. Line 28: Consider re-writing sentence starting at Laboratory and ending in treatment. The intended meaning is not clear. Specifically, what do absorption buffer refer to?

Good point! We changed line 28 to read: 'When forming a biofilm, OG1RF(pCF10) constructs small complex structures with variable cellular packing. Laboratory experiments, detailed below, suggest that the presence of these preformed structures, in which aggregated regions form protective walls that enclose less densely packed areas. These aggregated structures surrounded by lower density biofilm are shown to be a necessary condition for sustained biofilm growth and protection during erythromycin treatment' (now lines 35-40).

2. Line 48: In contrast to what?

This contrasts with the previous sentence. Line 48 from initial submission's prepositional phrase is edited (now line 58).

3. Line 64: What is the necessary and sufficient statement?

Line 64 of original submission (now line 80) was clarified to read 'We determined that an adequate eDNA cloud covering and initial structure formation is both necessary and sufficient for the biofilm's survival under erythromycin treatment.'

4. Line 75: Here would be a great place to explain exactly what the "demonstrated rapid one hour remodeling" is, providing a figure and accompanying diagram highlighting the specific characteristics that your model is looking to reproduce or explain.

This issue should be resolved now that the results precede the methods section.

5. Line 77: How does 2D capture the spatial heterogeneity of the biofilm? Can you please describe this heterogeneity? Please define which coordinates are being represented (xz), this information is provided later but it is confusing at this point. Could be part of the diagram suggested in comment

These are good questions! The heterogeneity of the biofilm's cellular density can be seen clearly in orthogonal (y-z) slices as seen in figures 2, 3, 4, 5, and 10. But it is correct to assume that a 2D image cannot capture the complete picture of structural composition. To ensure that we were capturing most of the critical structure during this modeling process, we did assess whether, given a protective environment in the y-zone direction, you could assume it also exists in the x-z direction. This radial symmetry turned out to be an appropriate assumption after this analysis. To clarify this assumption, we added lines 131-135 reading: 'Prior to constructing the model in two dimensions, we assessed the structures for radial symmetry of protected regions which was well satisfied. We conducted this assessment by performing the image processing routine on x-slices and comparing them to the corresponding, intersecting, y-slices. This symmetry is discussed further in Sec. 4'

Additionally, this work is intended to then transition to a PDE model where more complex 3D simulations can be carried out (ongoing research).

6. Line 82: As I first read through, I was confused by the sentence "stressed cells require less room to divide". I think this would be fixed by addressing comment 4, but overall please justify why "stressed cells require less room to divide", and how "this difference in cellular division captures the biological process of aggregation".

Further explanation has been added between lines 82-83 of initial submission (now line 294). Essentially aggregation substance is a sticky, large surface protein that binds cells to one another. Thus, when a cell expressing aggregation substance divides, it does not completely detach from the newly formed cell. Thus, the cells remain in a closer proximity than under normal conditions.

7. Line 86: Please address your choice of using cellular automata. Specifically, what are the advantages over a discrete agent-based model for example and why is this the correct model to represent the biofilm. Please provide possible limitations for your model.

Cellular automata and individual based models in general give the modeler unparalleled ability to capture fine-grain interactions between cells which is critical when exploring the cell-to-cell interactions that lead to a global response. Current work is being done to devise a continuum model, informed by the models developed in this paper, to run the simulations on larger, more complex domains. An explanation of why we use this model is included after line 86 of the original submission (now 306).

It reads: 'Previous attempts to directly construct a PDE model for bacterial growth with an associated stress response were futile as the cell-to-cell interactions were not well enough understood. Thus, we pivoted to using the framework of cellular automata which has given us unparalleled abilities to parse and validate cell-to-cell interactions that lead to the globally observed system behavior. The creation of this model is an important step towards constructing

a continuum model with the intention of running simulations on larger, three-dimensional computational domains.'

An additional statement was added after line 614 of the original submission (now 719) stating the prohibitive time cost of running larger simulations (particularly those in 3D). I also included that we are currently working on a continuum model which significantly reduces computational cost.

It reads: 'Mathematically, we are currently developing a continuum-level description of the cellular automata model to run simulations on larger, more complex computational domains. Cellular automata computationally scale poorly with increasing domain size, therefore a continuum model will be necessary.'

8. Line 96: Please justify your choice for only 2 stress states. Additionally, in the diagram in fig 1. if a stressed cell divides shouldn't the new cell also be stressed, e.g. a member (1,1)? In a sense this goes back to my question about choosing a binary definition of stress.

Good question! The induction of stress is biologically a cell's expression of aggregation substance. Aggregation substance is a sticky, large surface protein whose expression is an irreversible process. A more detailed explanation of this process is added between original lines 82-83 and new lines 293-298.

It reads: 'Biologically, the induction or switching from 0 to 1 of stress is the process of aggregation substance induction undertaken by the cell. Aggregation substance is a stable cell wall protein that binds lipoteichoic acid on adjacent cells and is an irreversible, discrete, and stable process [65]. A cell expressing aggregation substance is sticky and will not fully separate from any newly divided cell it produces thereby reducing the distance used for cellular division.'

9. Line 109: In definition 2, c is not defined anywhere in the text. Additionally, when presenting definitions please wrtie the abbreviation next to the plain English. For example: the interaction neighborhood (NL) of r for bacterial…

Updated Definition 2 accordingly!

10. Line 121: delete recall that, it is likely not common knowledge for all readers.

Corrected!

11. Line 153-155: please define decay. Reference to fig 5 is out of order, will probably be solved by comment 4. Furthermore, how is your assumption that decay rate and death rate are equivalent. Can you measure decay rates from your experiments to verify this assumption?

This is a good question! Decay rate is an unknown parameter, but for simplicity, we keep death rate constant across all intracellular antibiotic concentrations. The paper was corrected accordingly, see line 375.

12. Line 159: Would be good to move this to the description of your system in comment 4. It is a bit strange to specify micrometer units for L, without providing a typical numerical range.

L is quantified in Section 3.4. Although mathematically all coefficients must have units even before prescribing a precise value since we know L is a length and the length scale used for the model is micrometers. Moving models/methods to after results should have resolved this concern.

13. Line 163: Please justify why 8 positions is sufficient and why diagonals or other possible configurations are not considered.

Good question! These 8 positions are the only positions (in the entire lattice) that are 5 μm or 1 μm away from the given node. Without this restriction, a newly divided cell could break the carrying capacity law of the local grid. A clarifying sentence has been added directly after line 163 of the original submission (now line 381).

It reads: 'Looking at the neighborhood, N_L({\Vec{r}}), because a cell must grow either 1 or 5 micrometers apart, we find that a bacterial cell can only propagate from 8 positions: those that are adjacent to the cell and those at length L away as illustrated in Fig. 9A. This choice of using these positions is consistent with the use of two five-point stencils for numerical discretization: one for the average growth distance under stress and one for the average growth distance not under stress.'

14. Line 177: Can you please explain why assuming a buoyant force makes sense in this system? One might think that capillary forces from the water and adhesion forces from the ECM would penalize out of plane growth and prefer side motion instead.

Good question! A growth anisotropy was observed during biological experiments and in the microscopy data, but because we cannot confirm where exactly this growth anisotropy comes from, we edited the statement to be more general. This growth anisotropy is a commonly found feature within biofilms but it remains unknown whether this is due to a buoyancy force or to optimize nutrient access. Lines 179-178 of the original submission were edited to reflect this (now line 402).

They now read: 'During biological experiments, we noticed a growth anisotropy. The bacteria within and around the complex structures tends to favor growing up/down. This is a well-documented phenomenon that can be attributed to either optimizing nutrient access [34] or a force of buoyancy [69]. To take this growth anisotropy into account, we say that if the bacterial cell located in \Vec{x} has the space available to divide up and down, it will do so.'

15. Fig 2. Caption "On the other hand, if r is to the left or right of x and if lattice nodes y2 or y3 are empty then x will not place the new cell in r. x will instead place the cell in y2 or y3 due to the force of buoyancy." Are these labels correct? Should it be y1 or y2?

Yes, thank you! This has been corrected.

16. Line 183-192: Explain in plain English these probabilities please. Maybe consider simplifying the mathematical definitions to plain English and presenting the full mathematical framework in the SI.

Moving the models/methods to after the results should avoid the need to place these definitions in an appendix. A clarifying statement prior to line 183 of original submission was added (now line 408).

It reads: 'Therefore, we must have separate growth probabilities for up/down and left/right growth with a further separation of the division distance. With all this in mind, we can formulate the following separate growth probabilities that a cell in \Vec{x} with a corresponding division distance divides and places the new cell into an empty lattice node, \Vec{r}, which is left/right or up/down from\Vec{x}$'

17. Line 220: "It is known" please provide citation.

This has been corrected to explain our reasoning: 'Because the amount of initial rigid structure is constrained by factors other than nutrient availability and continues to increase during the three days of prior biofilm growth, stress by direct contact must occur on the same time scale as the formation of initial complex structures (days).' This is now on line 449.

18. Line 221: First time that a rapid increase in bacterial population is mentioned, refer to comment 4.

This is addressed by moving results to before models and methods.

19. Definition 7, same as before consider using plain English to explain the assumptions here. Please explain how ψ and $\psi 2$ are determined.

Definition 7 has been altered to explain the reasoning of equations.

20. Line 249, equation 10: Why is the probability of a cell being at stress 0 and remaining at stress 0 is equal to having a space remain empty?

Thank you for pointing that out, it has now been corrected in the manuscript! Although this probability is not used during the actual numerical simulations, we did have a typo (0 to 0 instead of 1 to 1).

21. Fig 3: Consider providing this diagram earlier, maybe as part of figure 1 explaining the experimental results and your proposed model.

Good question! The lattice and the computational domain, although related, are two separate entities. A clarifying statement was added after line 226 in the original submission (now line 489).

'Note that this is the computational version of the previously defined lattice, \mathcal{L}.'

22. Line 270: are cells non motile? Or can they move to other parts of the biofilm?

Good question! Cells are non-motile, and to make this clearer, a sentence has been added line 143 of the original submission (now line 386).

It reads: 'An important biological fact, used as the basis of this model, is that these cells are non-motile, and therefore biomass evolution over the simulation occurs via growth, not movement.'

23. Line 282: provide citation for Todd-Hewitt broth.

Citation has been added!

24. Line 287: Define MIC

MIC (minimum inhibitory concentration) acronym now written out when first mentioned in the paper.

25. Table 1: Please provide variable names in plain English to refer to as reading the paper. Ta: how is this estimated?

Fixed by providing a comprehensive table of definitions. T_a is just 1X MIC, value now stated in the table.

26. Line 322: Provide definition of CFL acronym.

Acronym spelled out now (Courant-Friedrichs-Lewy).

27. Line 373: How do you know that cells in protected regions are unstressed? I would suggest using a stress reporter on the experimental setup to verify this assumption if not done already.

In the model a "stressed" cell is defined as expressing aggregation substance, which can be initiated by 3 different types of stress signals; antibiotics, contact with the aggregate, or the pheromone cCF10 signaling pathway. "Unstressed" cells are not expressing aggregation substance and may be present in multiple locations including low density biofilm. We consider this a low stress environment because it is present throughout untreated biofilms and only in protected regions of treated biofilms. In response to comment 48 that also mentioned reporters,

we have found that expression of reporters such as GFP and mCherry slow doubling time by 30% and significantly interfere with the rate of structure formation and remodeling, potentially due to energy drain of expressing the fluorescent protein.

28. Line 407-411 I don't understand this, can you please provide a diagram of the experimental setup to understand what is causing the described shift? Why is the system not allowed to equilibrate before imaging?

This statement has been clarified in Section 3.6.2 and now reads:

'Plasmid-free *E. faecalis* OG1RF biofilms consist of a homogeneous densely packed rigid base overlaid by a very low-density biofilm matrix. This upper layer exhibits notable movement whose biophysical basis is not yet completely understood [78-79]. The microscope used in these studies is a Leica SP5, so the imaging time of a 3D biofilm is about 15-20 minutes. The cell movement in the lower density regions of the biofilm is rapid (most commonly 0.06-0.10 μm/s resulting in movements of 2-10 μm^3) [78]. Therefore, the inherent movement in the lower density biofilm, coupled with the slow imaging time of the microscope, leads to blurring of the individual cells in the lower density biofilm, yielding a low signal intensity. This low signal intensity is thresholded when characterizing aggregated structures, which are accurately quantified at high signal thresholds. It is therefore necessary to reintroduce the less densely packed biofilm uniformly for simulation. The individual cells that can be seen in the lower density biofilm are spaced approximately 5 μm apart (Fig. 10), so reintroduction involves filling in the less densely packed biofilm at a distance of approximately 5 μm. The result is an initial condition with defined preformed complex structures and surrounding less densely packed cells.'

29. Line 421: In the current model only xz slices are considered, but does the region need to be protected in both x and y? I don't see islands of less-densely packed cells amongst dense structures in many of the images in Fig 5.

See response to comment 14.

30. Lines 429- 438 do not belong in the results section.

Agreed, these lines have been moved to the introduction.

31. Fig 5 caption: The number 72 doesn't add up. 12 x 3 replicates is 36?

Yes, thank you! This has been corrected (+3 for OG1RF biofilms).

32. Fig 5 and Line 449: before treatment there is a uniform lawn of cells, after treatment there is clear regions that no longer have cells, what happens to the cells in those regions? The increase in CFU might be related to relocation of the cells in those regions. Why are you confident that the treatment of Erythromycin does not affect the properties of the ECM such that the CFU counts are higher because it is easier to dissociate the structures, leading to more individual bacteria in the serial dilutions? Please provide a control image showing what a biofilm without pCF10 looks like before and after treatment, also please provide a control image showing what a treatment with only media looks like.

When counting cells the structure is disrupted by sonication. The biofilms are grown on circular coverslips of the same size and composition as the bottom of the optic bottom plates. The biofilms are washed, 500 microliters of PBS is added to the wells. The coverslip and PBS containing any dislodged biofilm are added to tube and the cells are disrupted by sonication. Empirical experiments were done to establish the settings that would disrupt the entire biofilm by inspecting the sonicated material and coverslip fragments by microscopy. Sonication of planktonic cells, which are unprotected by any clumping, at the same setting did not decrease the CFU, confirming the setting was killing any cells. As such, the CFU is a measure of the bacteria in the entire biofilm, rigid base, low-density viscous and complex structures. Dispersal

and reorganization of the existing biofilm bacteria would not lead to a two-fold increase in number.

It is formally possible that the lower CFU in the untreated biofilm was due to dispersal of the biofilm that halted under antibiotic selection although to date we are not aware of a dispersal mechanism in *E. faecalis* biofilms. This is now noted on lines 65 and 120 of the manuscript.

Further, we are unable to provide a control image of the same biofilm before and after treatment because the 15-20 minutes required for imaging an entire biofilm with our Leica Sp5 may significantly change the ability of the biofilm to remodel within 50 minutes.

33. Line 445: "One can see that the amount of preformed .." As far as I can see, this is not shown, as preformed structures are not quantified anywhere.

Quantitation of the size of the aggregated regions within the complex structure has been added to Fig. 5 (now Fig. 1).

34. Lines 451-453: please provide an image or diagram explaining this.

See response to comment #28.

35. Line 464, make a reference to each individual panel at the different steps in the workflow please.

Great suggestion! We made references to the individual panels.

36. Fig 6.E, explain what the different colors in this image represent. Overall this image is missing labels and a clear description.

Good point! Labels for the two clusters have been added to the image.

37. Fig 6.F Significance is provided with ** but caption doesn't specify what ** represents. Would be good to provide a panel with untreated and treated images in the supplement so the reader can visualize the difference in the protective volume.

Definition of double star significance added to caption of figure 2F and reads 'Double star significance indicates a p-value < 0.01.' Untreated and treated images can be seen in Figure 5 and Figure 1.

38. Fig 6.G Please provide an example of how the protective region structure volume was calculated.

Volume computation is clarified in list of image processing procedure on original submission line 483 (now line 157). It states, 'If a protective region is determined to exist for the given x-slice, the volume of each cluster (i.e. the number of cells contained in each individual cluster) is stored.'

39. Fig 7, line 503-504. How do you know this is the eDNA cloud? Was the eDNA labelled separately? If not please justify or provide new labelled eDNA data.

Good idea, see modified Figure 3!

40. Line 510: Overall, if parameters such as γp are to be used far from their definition, please remind the reader what they stand for (or organize them all in an easy to reference table. See comment on table 1)

Added a table of definitions!

41. Line 521: Why does this boundary condition result in no flux through $\partial\Omega 1$?

In this scenario all antibiotics enter the computational domain from above (since no eDNA barrier is introduced for this test). This is commonly termed a 'bulk boundary condition'.

42. Fig 8. Why is B not the same height (z) as A? It seems as if the biofilm is cropped?

Good question! A clarifying statement was added to the caption of figure 8 (now figure 5) noting: 'In order for the cells to respond to antibiotic influx an extended vertical domain is required, since the stress distance (μ_d) is 15 μm. Therefore, we added an extra 2 layers of uniformly distributed cells with a non-stressed carrying capacity above the complex structures as is seen in microscopy images.'

43. Combine lines 534-536 and lines 543-544?

The lines have been combined.

44. Please discuss if assumptions in your model, such as the buoyant force and limited motion of the cells (8 positions) could be responsible for the observed results in Fig 8.

Regarding the 8 positions, see also response to item 13. Regarding buoyancy, there is no limitation to grow side to side as long as vertical growth is not possible (due to occupancy). This condition is needed to account for the experimentally observed growth anisotropy. Hopefully this is further clarified by sentences added after line 163 of the original submission (now line 402). The only assumption/observation, aside from established biofilm model assumptions is the growth anisotropy which is further explained in comment 13).

45. Line 550- 552. This is not clear, please explain.

When conducting the initial image processing, the density of each layer was computed. The cutting point of rigid base vs non-rigid base of the biofilm was computed from this parameter. Because we are now only working with 2D slices, this is no longer a global parameter. Around the complex structures, there tends to be less rigid base, therefore the same 'cut off' density leads to larger computed complex structures. But this is not a critical point, the doubling after treatment is what we are aiming to recapture.

Lines 550-552 (now line 251) were now modified to read: 'It should be emphasized that, since we are only working with a two-dimensional slice, we do not have access to any global parameters of the three-dimensional configurations. Without the global density parameters used in the removal of the rigid base layer, the volumes of the protective structures will typically be larger. Eight slices were randomly chosen, each from different untreated biofilms, such that they contained a protected region according to the previously defined image processing algorithm'

46. Can you estimate "CFUs" from your simulation results and match the doubling of cells in the experiment?

The simulated domain is just a subset of the entire 16 mm culture plate well therefore, the CFU cannot accurately be estimated from the simulation results. However, the doubling in size of the structures suggest similar behavior within all structures capable of continued growth could account for the CFU increase.

47. 577: Please explain why eDNA is not enough to protect WT E. faecalis. E.g what is the role of plasmid pCF10 in eDNA production. Without a control on WT, it's hard to interpret your results.

Good question! WT control has been added (see Figure 1). Additionally, a statement has been added after line 690 of the original submission (now line 676) stating: 'It is important to note that although eDNA is present in OG1RF *E. faecalis* without pCF10, it is not fully protective (Fig. 7). The eDNA cloud acts as a stop gate and as a result the local density of the biofilm increases prior to antibiotic penetration. Without the pCF10 plasmid, there is no aggregation response. So, any advantage provided by an extended initial antibiotic exposure period would not allow for the same reconfiguration response and, therefore, would not provide the wild-type bacteria with the same protections.'

48. Line 591: Why is it not possible to use a constitutive reporter to label the cells, such that staining is not required? You state that the reporters affect the growth rate, but is this a big effect? Or is it a result of phototoxicity?

Yes, it is quite an extensive effect. And since this is a growth dominated process, it greatly affects the structure reconfiguration response. A clarifying comment has been added to the discussion section around line 683. It states: 'Further, we found fluorescent reporters mCherry and GFP slow bacterial growth by a factor of 30% and reduces initial formation of rigid structures by 24 hours (data not shown) which prohibits their use in this case as the rapid reconfiguration is a growth dominated process.'

49. Please discuss other possible mechanisms other than eDNA that could lead to similar results

A line (716) in the discussion has been added stating: 'Erythromycin is not known to bind polysaccharides such as rhamnose, glucans, galactose, and N-acetylglucosamine in the ECM of *E. faecalis* [79]. If eDNA is found to not contribute to the overall resistance, although unlikely, it is possible that changes in the charge or packing of the EPS matrix due to aggregation accounts for the effect.'

Reviewer #3

We thank the reviewer for their helpful comments! We have modified the script accordingly in the following ways:

* The authors convincingly demonstrate that initial biofilm structure alone is not sufficient for survival. However, the argument that an eDNA cloud is sufficient seems more speculative. While eDNA clearly plays an important role, it is unclear whether it alone can ensure survival. Have the authors tested conditions where biofilms with eDNA but without complex preformed structures exhibit similar survival? If not, it may be more accurate to state that eDNA is necessary but not necessarily sufficient.

We have clarified the necessary and sufficient condition on line 64 of original submission (now line 80) to read 'We determined that an adequate eDNA cloud covering and initial structure formation is both necessary and sufficient for the biofilm's survival under erythromycin treatment.' An adequate eDNA coverage implies an adequate cover above complex structures and this (along with the initial structure formation) is the necessary and sufficient condition. We have additionally added images in figure 3 showing that the observed clouds above the complex structures are indeed composed of eDNA. In addition to this we added evidence and clarification statements and added other options instead of eDNA that could account for this overhead protection needed. The lines are found in the discussion (line 718) and reads: 'Erythromycin is not known to bind polysaccharides such as rhamnose, glucans, galactose, and N-acetylglucosamine in the ECM of *E. faecalis* [79]. If eDNA is found to not contribute to the overall resistance, although unlikely, it is possible that changes in the charge or packing of the EPS matrix due to aggregation accounts for the effect.'

* The definition and role of the stress variable in the model are unclear. While bacterial cells and antibiotic molecules are well-defined, 'stress' seems more abstract. What biological process does this variable represent? Does it correspond to a measurable quantity, and if so, what are its units? Additionally, the way stress influences bacterial growth and division appears to overlap with the direct effects of antibiotic exposure—would it be clearer to model growth inhibition as a function of intracellular antibiotic concentration instead?

Stress in this paper is defined as the expression of aggregation substance whose biological process is irreversible and discrete (see modified lines 293-298). Induction of our variable stress (going from 0 to 1) can be enacted in three ways:

1. **Intracellular antibiotic concentration exceeds a threshold (T_S)**
2. **Direct contact stress (a cell not expressing aggregation substance encounters a cell expressing aggregation substance)**
3. **Signaling molecules in local area exceeds a certain concentration (iCF10/cCF10 pheromones). The density and proximity of cells expressing aggregation substance is used as a proxy for the concentration of these signaling molecules.**

Furthermore, growth inhibition is already a continuous process modeled by a Hill equation and modulated by the intracellular antibiotic concentration. A clarifying statement pertaining to the function, f, being continuously dependent on the intracellular antibiotic concentration has been added after line 170 of the original submission (now line 393).

It reads: 'The function f is a Hill function that is continuously dependent on the antibiotic concentration, a, until a >=Tg at which point f =0'.

* I am unsure why the authors model erythromycin action as a threshold-based effect rather than a continuous dose-dependent response (line 122). Given that erythromycin is bacteriostatic, one would expect a gradual inhibition of protein synthesis rather than a strict transition from unstressed to stressed at a defined antibiotic concentration. Can the authors clarify the biological reasoning behind this

assumption? Would a graded response better capture the heterogeneity of bacterial stress responses under antibiotic pressure?

Yes, growth inhibition is a continuous process modeled by a Hill equation, but the expression of aggregation substance is a discrete process which is why there is a threshold for exhibiting a stress response. A clarifying statement pertaining to the function, f, being continuously dependent on the intracellular antibiotic concentration has been added after line 170 of the original submission (now line 393).

It reads: 'The function f is a Hill function that is continuously dependent on the antibiotic concentration, a, until a >=Tg at which point f =0'.

Another statement clarifying the decision to make the stress induction a binary switch has been added between added between original lines 82-83 and new lines 293-298.

It reads: 'Biologically, the induction or switching from 0 to 1 of stress is the process of aggregation substance induction undertaken by the cell. Aggregation substance is a stable cell wall protein that binds lipoteichoic acid on adjacent cells and is an irreversible, discrete, and stable process [65]. A cell expressing aggregation substance is sticky and will not fully separate from any newly divided cell it produces thereby reducing the distance used for cellular division.'

* The assumption that erythromycin halts both growth and death seems biologically unrealistic. While erythromycin is bacteriostatic and inhibits growth, cell death can still occur due to factors such as metabolic stress, prolonged inhibition, or cell lysis. The distinction between 'death' and 'decay' is unclear—what exactly does decay represent in the model? If decay is functionally equivalent to death, does the model account for varying survival rates within biofilm subpopulations? A more biologically realistic approach might include a low but nonzero death rate that accounts for metabolic failure or prolonged antibiotic exposure.

Thank you for the suggestion. We now keep death constant regardless of intracellular antibiotic concentration. Lines 151-156 from the original submission were removed and lines 149-150 were modified to read (now line 375):

'where gamma_d is the probability a cell will die within the time step dt which, unlike growth, is not dependent on the intracellular antibiotic concentration.'

This alteration does not change the simulations or end results. Also, 'decay' was removed from various sections of the paper and now just notes the cellular process of death.

* As far as I can tell, the model is implemented in two dimensions. Given that biofilms are inherently three-dimensional, can the authors comment on how this limitation might affect their results? For example, does the model adequately capture antibiotic diffusion and eDNA-mediated protection in a way that would still hold in 3D? If so, what justifies this assumption? If not, what key insights might change in a full 3D model?

This is a good point! We did check for radial symmetry of protected regions before developing the model in two spatial dimensions. A clarifying line was added (see line 131)

It reads: 'Prior to constructing the model in two dimensions, we assessed the structures for radial symmetry of protected regions which was well satisfied. We conducted this assessment by performing the image processing routine on x-slices and comparing them to the corresponding, intersecting, y-slices. This symmetry is discussed further in Sec. 4'

Thus, two spatial dimensions were adequate to capture the dynamics of the protected/protective regions.

* The model assumes that stressed cells grow at a distance of 1 μm apart, implying a reduction in cell size during treatment. However, I could not find experimental data supporting this assumption—does erythromycin exposure actually cause a physical reduction in cell size? If this is based on empirical observation, could the authors provide quantitative evidence (e.g., microscopy measurements)? If this is purely a modeling assumption, how do they justify it biologically?

Good question! This made us realize that we did not mention the size of the cells anywhere. Cells are roughly 1 cubic micrometer large, so growing 1 micrometer apart measured from center of cell to center of cell means growing directly adjacent to one another. No cell size reduction is necessary nor does it occurs. To clarify this, we added a sentence before line 347 of the original submission (now line 587).

It reads: 'The computational domain was discretized into lattice nodes of length 1 \mu m. Since bacterial cells are approximately 1 \mu m^3 in size, each lattice node can have either one cell or no cells inhabiting it.'

* The claim that CFUs double within an hour under erythromycin treatment is quite strong, especially given that erythromycin is bacteriostatic. The authors state that this increase is due to growth rather than movement or dispersal, but I am not sure the evidence fully supports this. Could they provide additional experimental validation to rule out alternative explanations such as biofilm dispersal, changes in culturability, or sampling variation? If direct evidence of growth (e.g., time-lapse imaging, biomass quantification, or DNA synthesis assays) is available, it would strengthen this claim considerably.

The CFU is a measure of the bacteria in the entire biofilm, rigid base, lower density biofilm and complex structures, therefore dispersal and reorganization of the existing bacteria would not lead to a two-fold increase in number. By quantitating the size of the aggregated structures before and after antibiotic treatment the size of structures (cubic micrometers) significantly increased (new data Fig. 3). Given that the aggregated structures account for most biomass, this suggests that the increase CFU was accounted for by the increase in the size of the aggregated structures. It is formally possible that the lower CFU in the untreated biofilm was due to dispersal of the biofilm that was stopped under antibiotic selection but to date, no one has identified a dispersal mechanism in *E. faecalis*. A statement has been added to the introduction to clarify we are not considering dispersal at this time because neither dispersal nor a mechanism for it has been described for *E. faecalis*. See line 65.

* The manuscript does not clearly define what 'untreated' means in the experimental setup. Were these biofilms grown in parallel under identical conditions, with the only difference being the absence of erythromycin? Were they sampled at the same time points? This is important for interpretation—if untreated biofilms also exhibit CFU increases, then the role of erythromycin in driving biofilm remodeling may be less clear. Can the authors clarify how the untreated control was designed and justify that it is a valid comparison?

The structures only grow very slowly with fresh media changes twice daily maintaining a steady state number in the biofilm (compare D1 vs D2 vs D3 T0 images) and their steady state numbers (T0 in the graph). Fresh medium changes (untreated) look the same as T0 biofilms. (T) See line 521.

* The existence of an eDNA cloud appears to be supported primarily by the microscopy image in Figure 7. However, it is unclear whether this is sufficient evidence to confirm both the presence and functional role of eDNA in biofilm survival. Could the authors provide additional validation, such as quantification

of extracellular DNA, DNase treatment experiments, or staining controls in untreated biofilms? Without these, it is difficult to determine whether the observed fluorescence represents functional eDNA rather than an artifact of cell lysis or antibiotic exposure.

Figure 7 (now figure 3) has been edited to show eDNA via propidium iodide staining thereby confirming the presence of eDNA in the clouds (new data). The determination of possible binding targets for erythromycin (rRNA) in the eDNA cloud and its contribution to protection using DNase may be complicated if ZDNA (not susceptible to DNase) is also present in *E. faecalis* biofilms. These experiments, however, are beyond the scope of this paper and are subject to ongoing research.

* The use of 100× MIC erythromycin seems quite high, and I could not find a clear justification for this concentration in the manuscript. How was this critical dose determined? Since biofilms are known to have diffusion barriers and antibiotic resistance mechanisms, what would be the expected erythromycin concentration inside the biofilm under these conditions? Have the authors considered performing a dose-response experiment to determine whether their findings are robust across a range of more clinically relevant antibiotic concentrations?

When studying antibiotic resistance in biofilms, the question is often how much resistance is actually conferred by this mechanism in comparison to an antibiotic resistance gene. Therefore, we chose 100X MIC as a dose to demonstrate how robust this system is in the protection of the cells from antibiotics. We do have data for 0.1 MIC, 1X, 10X and 100X, but preferred the high dose to highlight how robust the protection is against erythromycin.

* The manuscript would benefit from more consistent strain nomenclature. The first mention should use the full genus and species name (e.g., Enterococcus faecalis OG1RF), and subsequent mentions should abbreviate the genus (E. faecalis OG1RF). Additionally, bacterial names should always be italicized. Standardizing this throughout the manuscript will improve clarity and readability.

We apologize for the inconsistency in the strain nomenclature and the presence of italics on the genera and species name. This has been corrected throughout the manuscript *E. faecalis* OG1RF and *E. faecalis* OG1RF(pCF10) is now routinely used after the first mention of *Enterococcus faecalis*.

* The manuscript uses 'demonstrate' for both experimental (Line 29) and computational (Line 58) results. Since 'demonstrate' implies definitive proof, especially in mathematics, it may not be the best choice for computational findings.

This is a good point! ‘demonstrate’ has been changed to ‘suggest’ in each line.

* The manuscript provides a GitHub link for the code and supplementary videos, but the repository does not appear to contain any public files. Since the code is essential for assessing the computational model and reproducibility, could the authors ensure that the files are accessible to reviewers? If the repository is private, an alternative way to access the files would be helpful.

The code is available in the supplemental information submitted and divided by manuscript figures it produced. Additionally, a readme file was added to the supplemental information for further details about the code. Upon acceptance, the Github repository with the same files provided in the supplemental information will be made public.
