# Supplementary material for: Using stochastic cellular automata to model and define sufficient conditions for the survival of Enterococcus faecalis biofilms with the pCF10 plasmid under erythromycin treatment
Source: PLoS Comput Biol. 2025 Sep 2;21(9):e1013425. doi: 10.1371/journal.pcbi.1013425 (PMC12445747; doi:10.1371/journal.pcbi.1013425)
Supplement: Attachment — Submitted filename: Response_to_Reviewers_auresp_2.pdf [file pcbi.1013425.s039.pdf]

We would like to thank the reviewers for their thorough review of the manuscript.

1) Your manuscript's sections are not in the correct order. Please amend to the following order: Abstract, Introduction, Results, Discussion, and Methods

The order has been corrected.

Reviewers' comments:

Reviewer's Responses to Questions

Reviewer #1: From my point of view, the authors have addressed the points raised up in the first version/review adquately.
I could imagine some details to be done differently, but this is more a matter of taste.

A very little comment:
In Definition 7, it looks like something went wrong with the Math mode, could be corrected (no further review necessary for that).

Thanks for pointing that out! That seemed to be an artifact of the track changes document; the document without the track changes does not have this issue.

Reviewer #2: Overall, most of my questions were appropriately addressed in the manuscript. The restructuring of the results also helps in broadening the impact of the paper to a wider audience.
Some considerations that should be fixed.
- In line 111, there is a reference to Fig 1G-R, but the figure only has G panels.

Thank you for pointing out the typo! It has now been corrected.

- In section 2.3 a brief introduction of the cellular automaton model should be provided, to guide to reader in understanding the boundary conditions, and subsequent results. Ideally before line 193, as there is reference to sufficiency conditions in the model without having introduced the model. Which makes the paper read out of order.

A reference to the corresponding section outlining the model parameters was made to make the connection clearer.

- In line 277, this statement is very strong. Would recommend to switch to: Taken together our results strongly suggest that initial complex structures …

The sentence on line 277 was edited accordingly.

- In regards to line 283, and throughout the document, the word demonstrated usually implies mathematical derivation, or proven results in the literature, therefore when referring to experimental results it might be more appropriate to write experimentally observed. E.g. "capture the mechanisms pertaining the experimentally observed rearrangement of …"

The sentence on line 283 was edited accordingly.

- Include the variable a (100x MIC concentration) in one of the parameter tables.

Thank you for the suggestion! The variables a_1, a_2, and a_r have now been included in table 1.
